# Effects of muscle strength in different parts of adolescent standing long jump on distance based on surface electromyography

**DOI:** 10.3389/fphys.2023.1246776

**Published:** 2023-10-20

**Authors:** Yifei Wang, Delong Dong

**Affiliations:** Department of Physical Education, Ludong University, Yantai, China

**Keywords:** standing long jump, surface electromyography, linear regression, characteristics of muscle force, principal component analysis

## Abstract

**Objective:** To reveal the influence of muscle strength in different parts of the body on the distance of standing jump, to establish the key force phases of muscle strength in different parts, and to improve the recognition of movement norms.

**Methods:** VICON infrared three-dimensional motion capture acquisition and analysis system and Noraxon Ultium surface electromyography acquisition and analysis system were used to complete the surface electromyography signal acquisition of 18 randomly selected subjects performing standing long jump.

**Results:** 1) Triceps brachii, anterior deltoid, latissimus dorsi, semitendinosus, rectus femoris, upper trapezius, pectoralis major, and biceps femoris had significant effects on standing jump distance. 2) From the point of view of the key exertion phase of the standing jump mainly affecting the muscle group; the main exertion phase of the semitendinosus occurs from the rising stage to the descending stage; the rectus femoris, triceps, and latissimus dorsi occur during the ascending phase of the flight; the anterior deltoid muscle occurs in the transition stage from rising to falling in the air; the trapezius muscle occurs in the transition stage from pre-swing to kick-off.

**Conclusion:** 1) From the regression analysis of the measured muscles on the distance of each stage of standing long jump, deltoid muscle strength is conducive to the improvement of standing long jump distance, which further indicates the importance of upper limb deltoid muscle strength. 2) Through time series analysis, it is found that the force performance of the rectus femoris muscle at this stage can be used as the main identification parameter of standing long jump, and can effectively distinguish different types of movements.

## 1 Introduction

Standing long jump is a sport that starts from a standing position without any approach. It integrates physical qualities such as jumping, explosive power, body coordination and technique (Hraski et al., 2015). Generally speaking, the standing long jump reflects the level of human muscle power, which is related to the number of fast-twitch fibers and the cross-sectional area (Jian and Shengmin, 2019). The standing long jump is divided into four stages, which are pre-swing, take-off, airborne and landing. Among them, the swing power of the upper limbs, the kicking strength of the lower limbs and the core strength of the trunk will have a profound impact on the standing long jump. Standing long jump is a kind of coordination that requires the height of the upper body and lower body (Wakai and Linthorne, 2005), and it is completed by the swing of the upper limbs and the push and extension of the legs. Therefore, coaches and teachers should not only develop the lower body strength quality of students during training, but also the upper body strength of students. Many studies have investigated the role of arm movement (upper body swing) in standing long jump performance (Aguado et al., 1997; Ashby and Heegaard, 2002), and at the same time pay attention to the coordination and exertion of the muscles of the whole body during the entire technical movement. The standing long jump mainly focuses on feature recognition and training details. The contribution to the muscle strength of different parts of the human body and the key muscle strength indicators in different phases are not very clear.

The purpose of this study is to explore, through the collection of surface electromyography data, how to understand the force characteristics throughout the entire process of the standing long jump movement. This research aims to achieve a more accurate assessment of which muscles are more critical from a quantitative perspective and to better recognize and differentiate normative movements during different phases. This will enable effective diagnosis and objective evaluation, thereby diversifying research in the field of standing long jump. Ultimately, this research aims to provide quantitative references for jumping sports, contribute to the teaching of proper technical movements, enhance training effectiveness, and provide practical guidance for simulation and intelligent teaching.

## 2 Materials and methods

### 2.1 Test object

This study takes the five stages of the standing long jump, the pre-swing stage of take-off, the stretching stage of take-off, the stage of rising in the air, the stage of falling in the air, and the landing stage, as the research object. In China, the Junior High School Academic Proficiency Examination, commonly referred to as the “Zhongkao”, is an assessment that determines whether junior high school graduates have achieved the required academic standards. Physical education assessments are also included in this examination, and the standing long jump is one of the physical education test components. In order to ensure that students achieve the passing standards for the standing long jump, effective training is conducted. When selecting participants for the test, junior high school students who had been practicing the standing long jump for 6–12 months were chosen. During a 2-week screening process, 20 individuals were initially selected, but 10 did not meet the screening criteria. Subsequently, a second round of selection was carried out with another group of 20 students, of which 12 did not pass the screening. In the end, 18 junior high school students were selected from four different schools.

The basis for the selection of test subjects in this study is: 1) The test subjects have no sports injuries in the past 6 months; 2) Can walk normally and each joint activity is normal; 3) No other health diseases, no drug dependence; 4) All parents have agreed to sign the volunteer consent form; 5) The data obtained from the test are valid data. Before the start of the study, all subjects signed the volunteer consent form, and the study was approved by the academic ethics committee of the school (approval number: LDU-IRB202106011), and finally 18 subjects were included, aged 13.4 ± 2.52 years, height 175.13 ± 5.88 cm, weight 72.73 ± 8.33 kg, the experiment was carried out in the biomechanics laboratory on the fifth floor of Yantai Sino-Russian Science and Technology Innovation Park.

### 2.2 Test method

**FIGURE 1:**
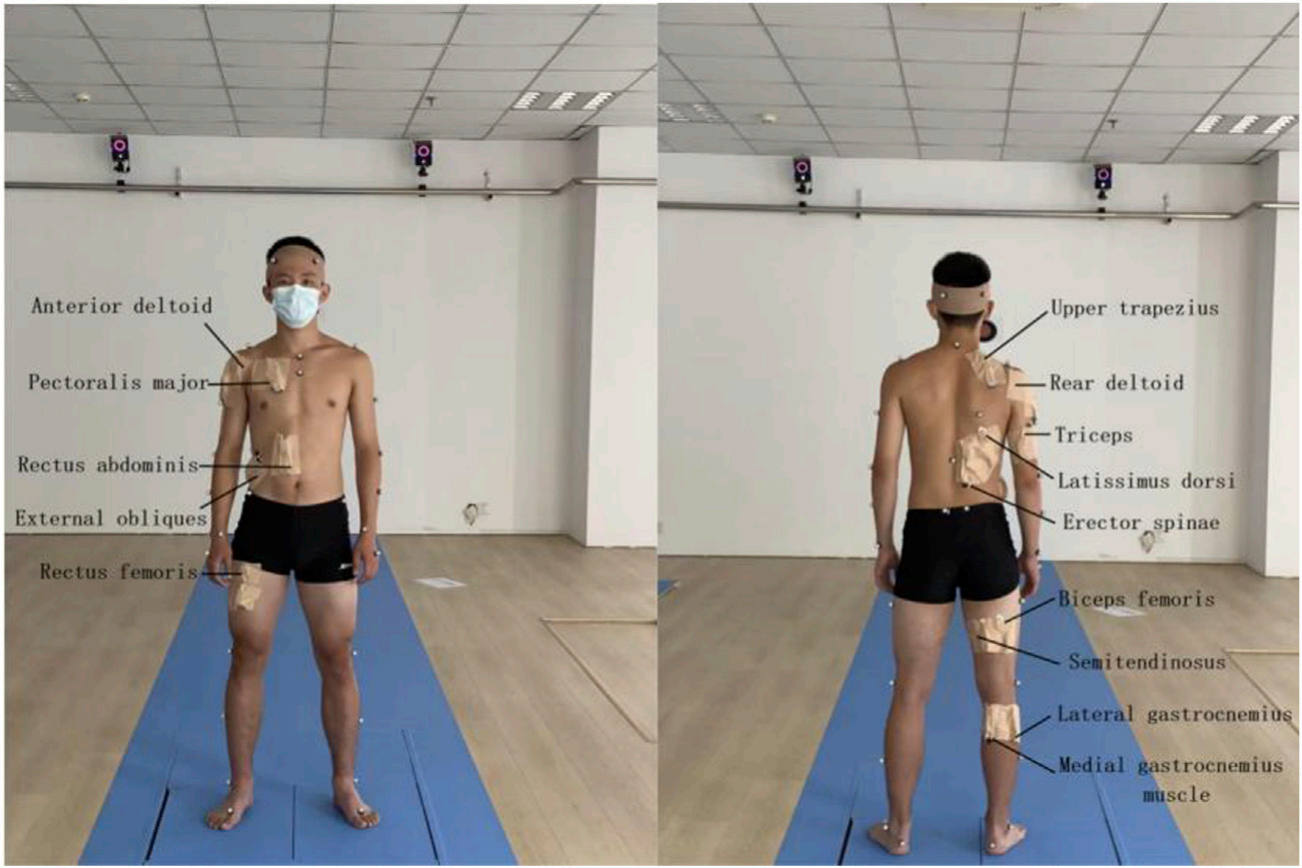
Schematic diagram of subject preparation and muscle names.

**FIGURE 2:**
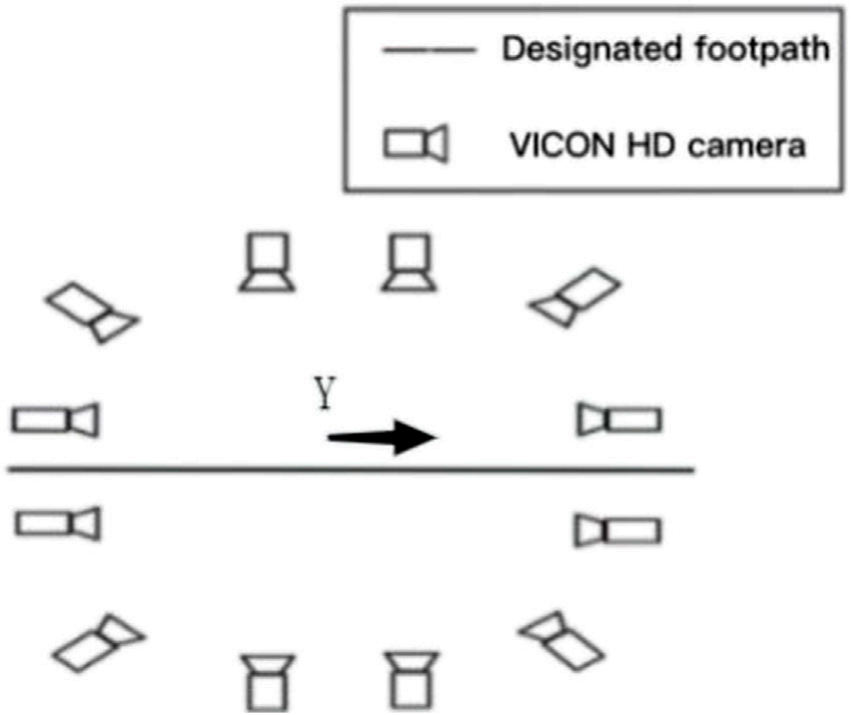
Schematic diagram of shooting position and coordinate axes.

In this test, VICON infrared 3D motion capture system and infrared motion capture HD camera (12 camera points, 120 Hz) were used to capture panoramic motion process and capture motion trajectory. Noraxon Ultium surface electromyography acquisition and analysis system (sampling frequency: 2000 Hz) was used to perform surface electromyography tests on subjects. Before the formal test, the organizer needs to conduct pre-test first, requiring the tester to be familiar with the whole process, conduct standardized operation of the instrument, prepare experimental consumables, select test objects that meet the requirements, and inform the subjects of the test process and other information. At the same time, the subjects’ height, weight, knee width, ankle width and other basic information were recorded and the informed consent was signed. Before the test began, the subjects changed their clothes and warmed up for 10 min wearing the elastic clothes prepared by the laboratory to practice the test movements and prevent sports injuries. During the preparation process, one operator will apply Mark points to the subject’s head, shoulder, hip, knee, and ankle joints, shave the hair at the point, and wipe the muscle abdomen of the test muscle with alcohol. After the alcohol was released, it was applied to the anterior deltoid tract, posterior deltoid tract, triceps brachii, pectoralis major, upper trapezius, rectus abdominis, external oblique abdominis, erector spine, lats dorsi, gluteus maximus, long head of biceps femoris, semitendinosus, rectus femoris, lateral gastrocnemius and medial abdominal muscles of gastrocnemius (Jinjian Huang, 2019), and fixed with skin film and adhesive materials, and the model was established (Figure 1). Before the test starts, the subjects are required to stand in the standing long jump preparation position, wait for the test personnel to give the start command, and make the test action, and complete three standing long jumps in total. The best results of the three tests are used as the research data in this paper. The subject jumped in the direction of the *Y*-axis (the dynamic shooting diagram is shown in Figure 2), measured the distance of the standing jump, checked the EMG data, and archived the data if it was valid.

## 3 Data processing

### 3.1 Phase division of standing long jump

**FIGURE 3:**
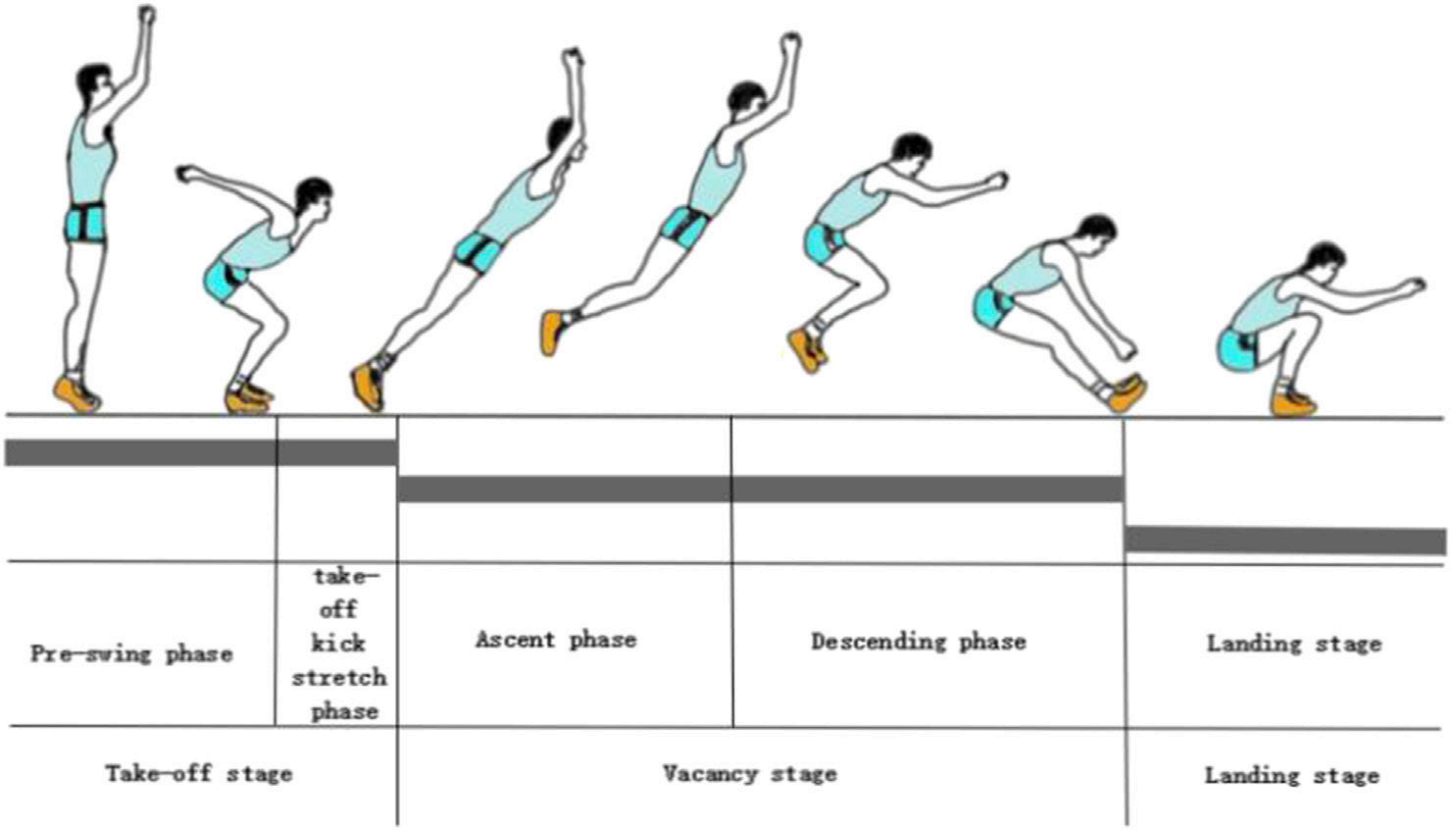
Time phase division of standing long jump.

In “Teaching Method and Training Method of Middle School Athletics Textbook”, the complete movement of standing long jump is divided into four stages, which are pre-swing, take-off, vacating and landing stages. However, the components of the standing long jump start at the very front of the feet before take-off and continue to the heels after landing. From this point of view, the whole standing long jump action can be divided into three stages, which are the take-off, vacating and landing stages. Wherein, the take-off stage includes the take-off pre-swing stage and the take-off kick-stretch stage, and the vacant stage includes the vacant ascending stage and the vacating descending stage. Therefore, in order to clearly analyze the myoelectric characteristics of standing long jump muscles, the complete standing long jump movement is divided into five stages, which are the take-off pre-swing, take-off kick extension, vacant rise, vacant descent, and landing stages (as shown in Figure 3), described as follows:

Take-off pre-swing stage: When the arm is pre-swing upward, the arm is pre-swing backward and downward while bending the knees for buffering, so as to minimize the center of gravity of the body.

Take-off kicking and stretching stage: After the center of gravity of the body is lowered to the lowest level, the body kicks off the ground until the moment when the feet leave the ground.

Ascending stage: until the center of gravity of the body is upward and at the highest position.

Flying and descending stage: the body’s center of gravity leaves the ground with both feet and begins to descend until the moment when the two feet touch the ground.

Landing phase: The vacating phase is over, the feet touch the bottom and the center of gravity of the body is lowered to the lowest position.

### 3.2 Types of standing long jump distance

In order to meet the needs of the following research, the standing long jump performance is divided into two categories according to the performance evaluation standards. Since the standing long jump reaches 250cm, it is full score (100 points), and the students who reach full score are few and nearly 80% of the students barely pass (Dongkui, 2022), so type I is the standing long jump score greater than or equal to 80 points, Type II is the standing long jump score less than 80 points. That is, the standing jump distance above 225 cm is Type I, and below 225 cm is Type II (see Table 1), which is convenient for the identification and comparison of different types of movements in this paper.

**TABLE 1:** Classification of different types of standing long jump for teenagers.

Coding	Standing long jump type	Distance (cm)
1	Ⅰ	278
2	Ⅰ	235
3	Ⅱ	183
4	Ⅰ	217
5	Ⅱ	180
6	Ⅱ	188
7	Ⅱ	236
8	Ⅱ	227
9	Ⅱ	150
10	Ⅰ	216
11	Ⅱ	158
12	Ⅰ	193
13	Ⅰ	192
14	Ⅱ	180
15	Ⅱ	184
16	Ⅰ	201
17	Ⅱ	189
18	Ⅱ	176

### 3.3 Myoelectric data acquisition

**FIGURE 4:**
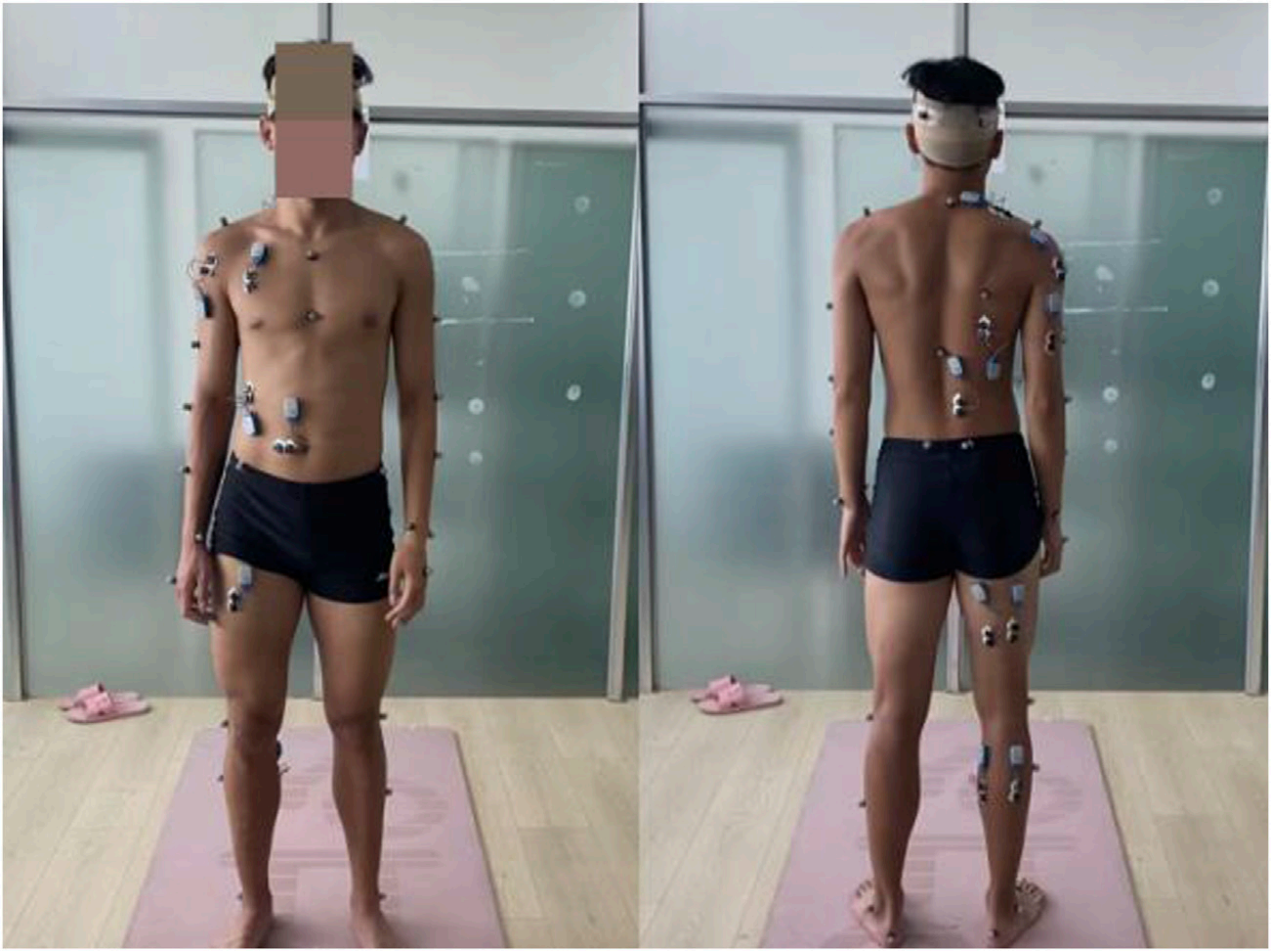
Electrode placement locations.

Utilizing the Noraxon surface electromyography device, position the electrodes longitudinally (along the fiber direction) on the muscles (Stegeman D & Hermens H, 2007) (as shown in Figure 4). This is done to collect electromyographic parameters of the muscles during the standing long jump motion. Import the tested original EMG data into the c3d format file, and use the MR3.16 software that comes with Noraxon to export the integral EMG data from the standard surface EMG report. Among them, the integral myoelectricity is usually defined as the absolute value of the range of the myoelectricity signal, which is related to the force sequence of the myoelectricity signal. It is usually used as the first index to be investigated clinically (see formula 1). In this study, relative electromyographic values, which are obtained by dividing the electromyographic values by body weight, are used to achieve greater precision. The larger the integral myoelectricity value, indicating that the degree of muscle force is greater.

$$IEMG=\sum_{i=1}^{n}\left|x_{i}\right|$$ (1)

### 3.4 Time series analysis

Time series analysis is mainly used to identify the muscle strength curves of different muscles through the way of time dimensionality reduction, which can be divided into several features, and determine which feature curves are more effective in recognizing different movement types, and further determine the key expression phase of a muscle in the whole movement process. The purpose of using this method in this study is to identify several curve features that each major influencing muscle can exhibit, which curve feature has more action recognition power (that is, it can be determined as a sensitivity index) and the key recognition phase in this curve feature on the basis of dividing different motion types of distance (specifically divided into two types of distance types type I and type II). This method was implemented in matlab 2021a by using FPCA toolkit of FDA (Ramsay and Dalzell, 1991). The 3rd-order Fourier basis was used to fit the surface EMG time series curves of muscles of different parts into functions (Donoghue et al., 2008), and the smoothing parameter was set to e−7. Subsequently, the dimensionality reduction of the function was decomposed into several principal components, with the cumulative contribution rate reaching 75% and the eigenvalue of each principal component greater than 50 (Ryan et al., 2006).

The specific formula is as follows:

$$\int a\left(s,t\right)\xi \left(T\right)dt=\mu \xi \left(s\right)$$ (2)

$$\omega \left(T\right)=\xi \left(t\right)\times \sqrt{v}$$ (3)

$$Y_{i}=\int \xi \left(t\right)x_{i}\left(t\right)dt$$ (4)

The a (s,t) in Eq. 2 is the covariance function of the function variable, ξ(T) is the eigenfunction, and μis the covariance matrix of the principal component; ɷ(T) in Eq. 3 is the weighted weight coefficient function of the principal component; Y_i_ in Eq. 4 is the score of the original function variable x_i_(t) on each principal component, and the eigenvalue corresponding to each principal component is calculated by the above formula, and then encoded in matlab 2021a to calculate the mean ± weight coefficient values.

The specific encoding is as follows:

for n=1:t

$$\text{if}\left(\text{MAXelement}\left(n,1\right)==1\right)$$

$$V1\text{value}\left(:,n\right)=\text{Resultdata}\left(:,n\right);$$

$$V1=V1\text{value}\left(:,\text{any}\left(V1\text{value},1\right)\right);$$

end

$$\text{if}\left(\text{MAXelement}\left(n,1\right)==2\right)$$

$$V2\text{value}\left(:,n\right)=\text{Resultdata}\left(:,n\right);$$

$$V2=V2\text{value}\left(:,\text{any}\left(V2\text{value},1\right)\right);$$

end

…

$$\text{data}1\left(n,1\right)=\text{meanValue}\left(n,1\right)+\text{meanValue}\left(n,1\right)*\text{MAXValue}\left(n,1\right);$$

$$\text{data}2\left(n,1\right)=\text{meanValue}\left(n,1\right)-\text{meanValue}\left(n,1\right)*\text{MAXValue}\left(n,1\right);$$

End

### 3.5 Mathematical statistics

After the extracted EMG data were entered and sorted out with Excel 2007, the regression model was hypothesized to be correlated with SPSS21.0, and the regression analysis model was established. See Formula 5, where X1,X2,X3, … X15 is the medial gastrocnemius, lateral gastrocnemius, semitendinosus, biceps femoris, gluteus maximus, erector spine, latissimus dorsi, anterior deltoid tract, pectoralis major, rectus abdominis, external obliquus abdominis, rectus femoris, posterior deltoid tract, triceps brachii and upper trapezius, β1, β2 … β15 was regression coefficient of medial gastrocnemius, lateral gastrocnemius, semitendinosus, biceps femoris, gluteus maximus, erector ridge, lats, deltoid anterior bundle, pectoralis major, rectus abdominis, external obliquus, rectus femoris, posterior deltoid bundle, triceps brachii and upper trapezius muscle in order. SPSS21.0 software was used to perform single factor ANOVA and LSD method was used. The significance level was set at 0.1 to analyze and compare the principal component scores of different types of adolescent surface EMG time series curves. Subsequently, independent sample *t*-test was adopted, surface electromyography curves were taken as independent variables, and different types of standing long jump actions were taken as grouping dependent variables. After screening out the muscle force sensitive indicators of standing long jump, Cohen’s d (Cohen, 2013) effect size (ES) was calculated. According to the threshold value described by Hopkins et al. It was divided into no effect, low effect, medium effect, high effect and great effect, i.e., ES < 0.20, 0.20–0.60, 0.60–1.20, 1.20–2.0, ES ≥ 2.0 (Hopkins et al., 2009). The integrated EMG growth rate, i.e., ΔIEMG, was calculated for muscle groups with no significant difference (Formula 3.8, ω is weight coefficient).

$$Y=\beta_{0}+\beta_{1}X_{1}+\beta_{2}X_{2}+...+\beta_{15}X_{15}$$ (5)

$$\Delta EMG=\frac{\left(M_{iemg}+M_{iemg}\times \omega \right)-\left(M_{iemg}-M_{iemg}\times \omega \right)}{M_{iemg}+M_{iemg}\times \omega }$$ (6)

## 4 Results and analysis

### 4.1 The influence of muscle strength in different parts of standing long jump on distance

The relevant data were input into SPSS analysis software, and the input method of parameter selection was used. After removing 7 parameters that could not significantly affect the total distance Y of the dependent variable standing long jump, the medial gastrocnemius, lateral gastrocnemius, gluteus maximus, erector spine, rectus abdominis, external oblique abdominus and deltoid fasciculus, In the final model, 8 parameters including semitendinosus muscle, biceps femoris muscle, latissimus dorsi muscle, deltoid anterior bundle, pectoralis major muscle, rectus femoris muscle, triceps brachii muscle and trapezius upper muscle were introduced. The results are shown in Table 2.

**TABLE 2:** Regression coefficient and significance test.

Parameter	Unstandardized regression coefficient	Standard error	Standardized partial regression coefficients	Regression coefficient test results	Significance level	Tolerance	Variance expansion factor
β0	1823.488	<0.001		<0.001	<0.001		
ST	1.699	<0.001	0.472	<0.001	<0.001	0.064	15.696
BF	0.106	<0.001	0.032	<0.001	<0.001	0.267	3.748
LD	−1.558	<0.001	−0.577	<0.001	<0.001	0.055	18.305
AD	0.770	<0.001	0.621	<0.001	<0.001	0.225	4.439
PM	−0.356	<0.001	−0.064	<0.001	<0.001	0.408	2.450
RF	0.710	<0.001	0.269	<0.001	<0.001	0.077	13.003
TB	−3.678	<0.001	−0.631	<0.001	<0.001	0.475	2.105
UT	0.732	<0.001	0.244	<0.001	<0.001	0.243	4.122

The absolute value of the standard coefficient of the 8 parameters is 0.631 > 0.621>0.577 > 0.472>0.269 > 0.244>0.064 > 0.032. It can be concluded that the contribution rate of these 8 parameters to Y is ranked as triceps brachii > deltoid anterior bundle > Latissimus dorsi > semitendinosus > rectus femoris > upper trapezius > pectoralis major > biceps femoris. The main factor affecting Y is triceps brachii, followed by deltoid anterior bundle, indicating that strengthening the training of deltoid anterior bundle can effectively improve the distance of standing long jump. Accordingly, the model estimation results can be obtained as follows:

Y=1823.488+1.699ST+0.106BF−1.558LD+0.770AD−0.356PM+0.710RF−3.678TB+0.732UT (7)

(Note: ST is semitendinosus muscle, BF is biceps femoris muscle, LD is latissimus dorsi muscle, AD is anterior deltoid tract, PM is pectoralis major muscle, RF is rectus femoris muscle, TB is triceps brachii, UT is upper trapezius muscle).

### 4.2 The distance of the standing jump mainly affects the phase recognition of the key exertion of the muscles

#### 4.2.1 Recognition of key phases of semitendinosus muscle exertion

**FIGURE 5:**
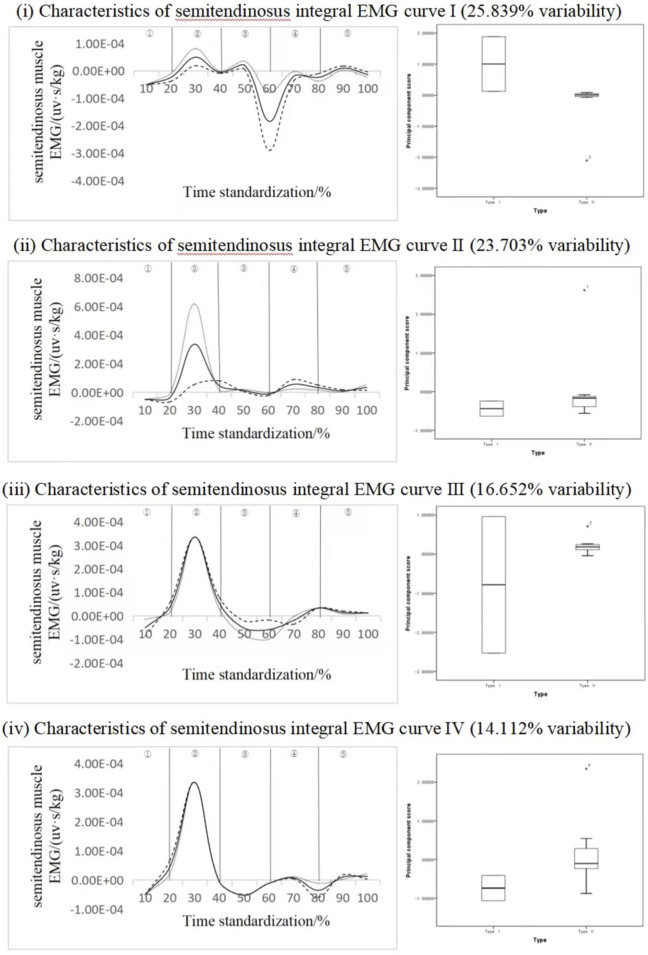
Mean ± weight coefficient of integrated EMG of semitendinosus muscle and principal component scores at different stages of motor development.

Figure 5 shows the dimensionality reduction results of principal components of the integrated EMG time series curve of the semitendinosus muscle, and the solid line in the figure is the curve of the mean change of IEMG. "--" means adding the multiple of the principal component weight on the basis of the mean; ″……″ On the basis of the mean value, subtract the weight multiple of the principal component, and the following number is the same (Yinshuang et al., 2017); Among them, ① is the pre-swing stage of take-off, ② is the stage of take-off and push and stretch, ③ is the stage of aerial ascent, ④ is the stage of aerial descent, and ⑤ is the stage of landing. The integrated EMG time series curve can be reduced into four principal components with characteristic values of 139.530, 127.996, 89.919 and 76.205, expressing variability of 25.839%, 23.703%, 16.652% and 14.112%, respectively. The cumulative contribution rate is 80.306%. The scores of all principal components of subjects with different motion distances did not have statistical significance, but according to the change curves of each principal component analysis, there were mainly three variation time intervals (20%–40%, 50%–70% and 70%–90% bands), especially the variation characteristics of principal component 1 and principal component 2 were more obvious. The variation fluctuation of principal component 3 and principal component 4 occurred significantly in the periods of 50%–70% and 70%–90%, respectively. Principal component 1 ΔIEMG% = 92.30%, principal component 2 ΔIEMG% = 91.26%, principal component 3 ΔIEMG% = 74.13%, and principal component 4 ΔIEMG% = 80.60%. From the point of view of the score of principal component 1, the score of the myoelectric principal component of the semitendinous muscle of type I adolescents was higher than that of the semitendinous muscle of type II adolescents. It can be considered that principal component one is the main curve feature of the muscle force, which can effectively divide different standing long jump action types (that is, the sensitive index of action type division), and 50%–70% action phase segment is the key force phase, and the muscle force at this stage can effectively identify different action types, which can reflect the different influence on the distance.

#### 4.2.2 Biceps key force phase recognition

**FIGURE 6:**
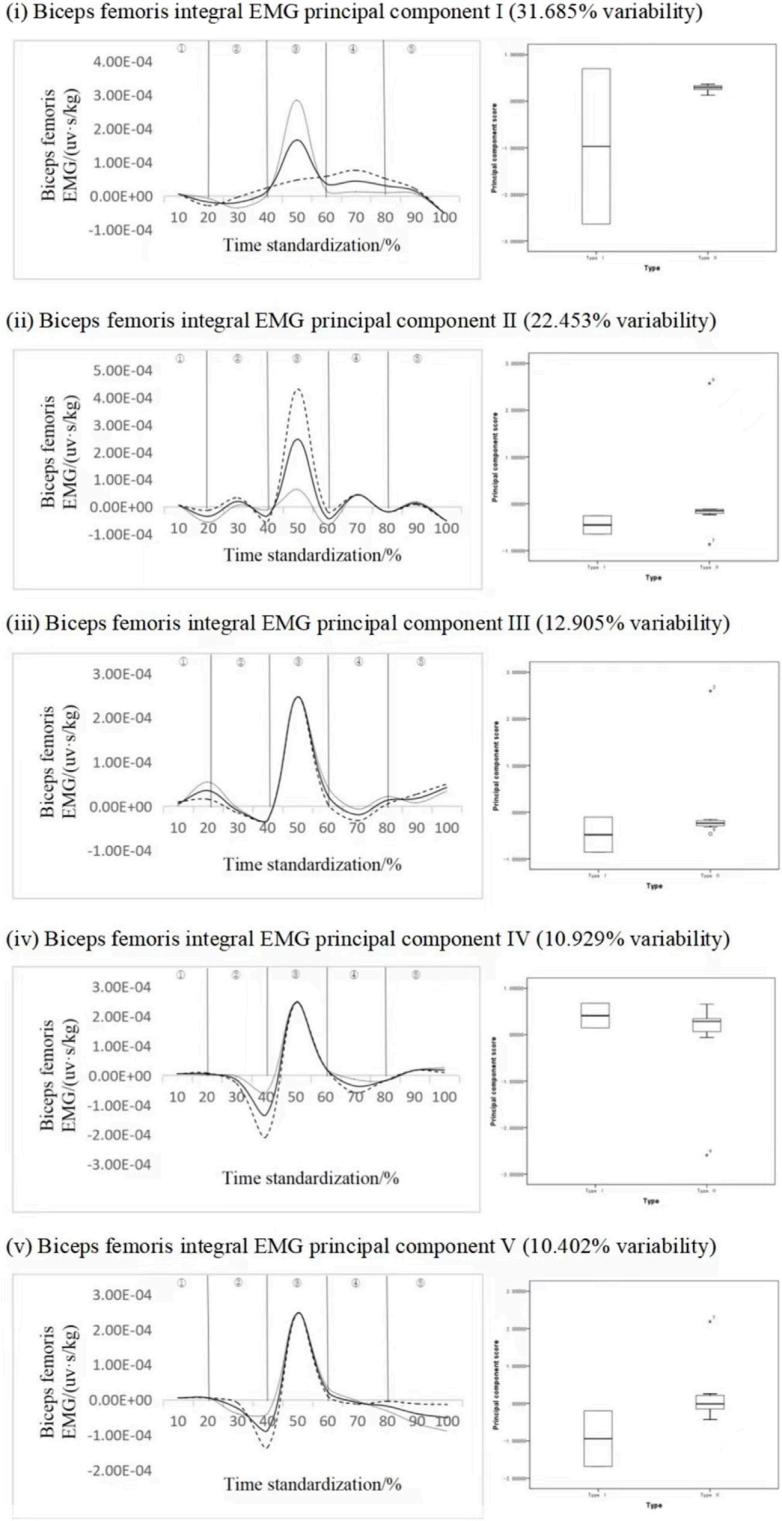
Mean ± weight coefficient of integrated EMG of biceps femoris and principal component scores in different motor development stages.

Figure 6 shows the principal component analysis results of the integrated EMG time series curve of biceps femoris muscle. The dimension of the integrated EMG time series curve is reduced into five principal components, and the eigenvalues are 171.098, 121.244, 69.689, 59.016, 56.172. The variation of 31.685%, 22.453%, 12.905%, 10.929% and 10.402% were expressed respectively, and the cumulative contribution rate was 88.374%. The results showed that the scores of all principal components of different types of subjects were not statistically significant, but from the analysis curve of each principal component, The variation time interval of principal component 1 and principal component 2 is the peak stage, the variation interval of principal component 3 is the first peak and second trough stage, and the variation interval of principal component 4 and principal component 5 is the trough stage, principal component 1 ΔIEMG% = 83.45% and principal component 2 ΔIEMG% = 85.32%. Principal component three ΔIEMG% = 79.15%, principal component four ΔIEMG% = 89.32%, principal component five ΔIEMG% = 76.01%, but there were no statistically significant differences in different types of standing long jump. On the whole, principal component 4 represents a greater myoelectric difference, and it can be considered that principal component 4 can effectively divide different movement types, and the key force phase occurs in the air ascent stage, which can reflect the influence of this muscle force on the distance of the long jump.

#### 4.2.3 Key force phase recognition of deltoid anterior bundle

**FIGURE 7:**
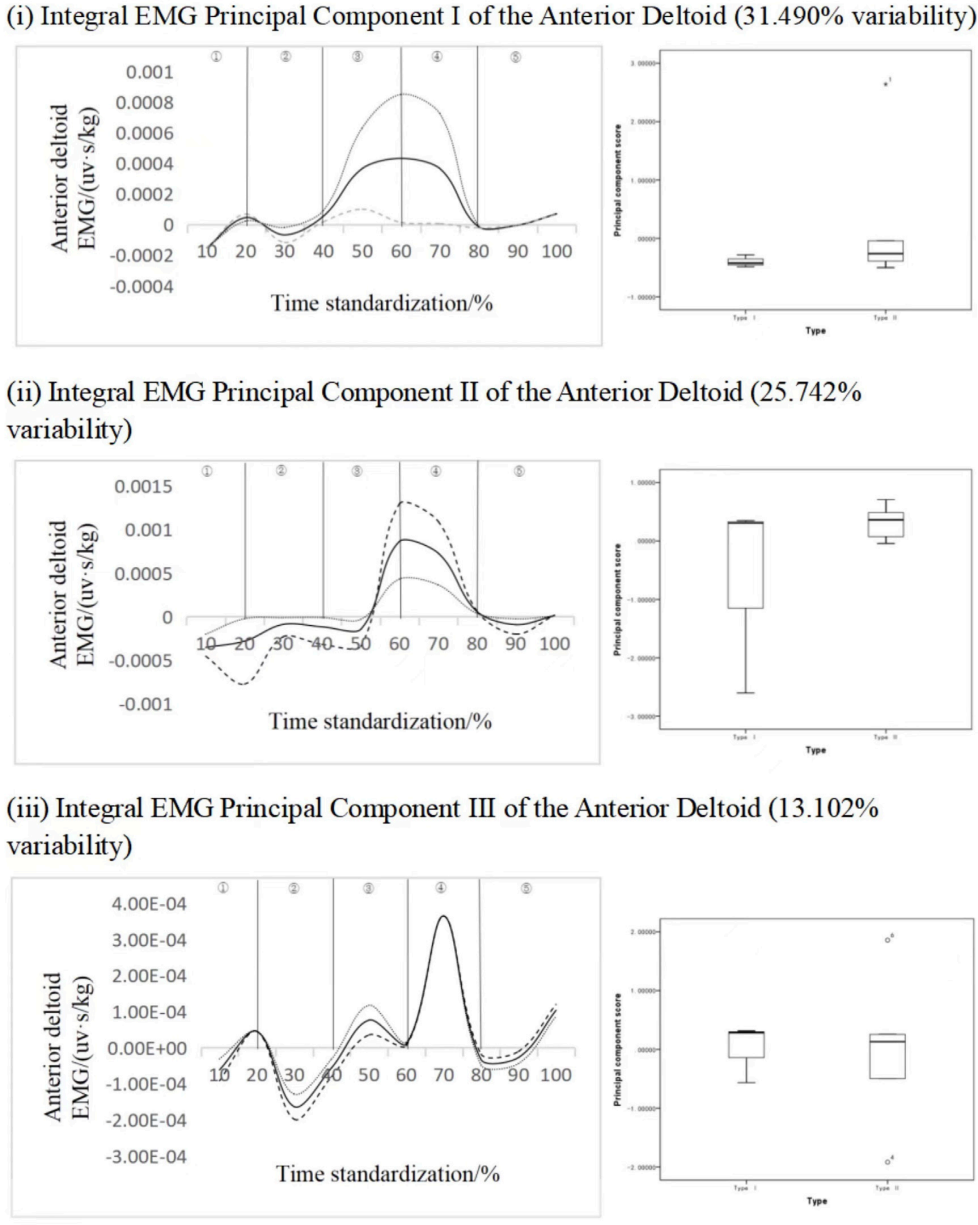
Mean ± weight coefficient of integrated EMG of deltoid anterior bundle and principal component scores of different motor development stages.

Figure 7 shows the principal component analysis results of the integrated EMG time series curve of the deltoid anterior bundle. The integrated EMG time series curve was reduced in dimension to three principal components with characteristic values of 170.043, 139.004, and 70.750, expressing variability of 31.490%, 25.742%, and 13.102%, respectively, and the cumulative contribution rate was 70.333%. The results showed that the scores of all principal components of different types of subjects did not have statistical significance, but from the change curves of each principal component analysis, the variation characteristics of principal component 1 was more obvious, and the variation interval of principal component 1 was in the period of 30%–80%, principal component 2 was in the period of 10%–50% and 60%–80%, and principal component 3 was in the period of 30%–60%. Principal component I ΔIEMG% = 99.19%, principal component II ΔIEMG% = 89.67%, principal component III ΔIEMG% = 69.79%. It can be considered that principal component 1 can effectively identify different types of long jump movements, and the key force phase is the transition stage from rising to falling in the air, indicating that the transition stage from exertion to relaxation of the deltoid anterior fasciculus has an important effect on the distance.

#### 4.2.4 Recognition of key force phases of rectus femoris

**FIGURE 8:**
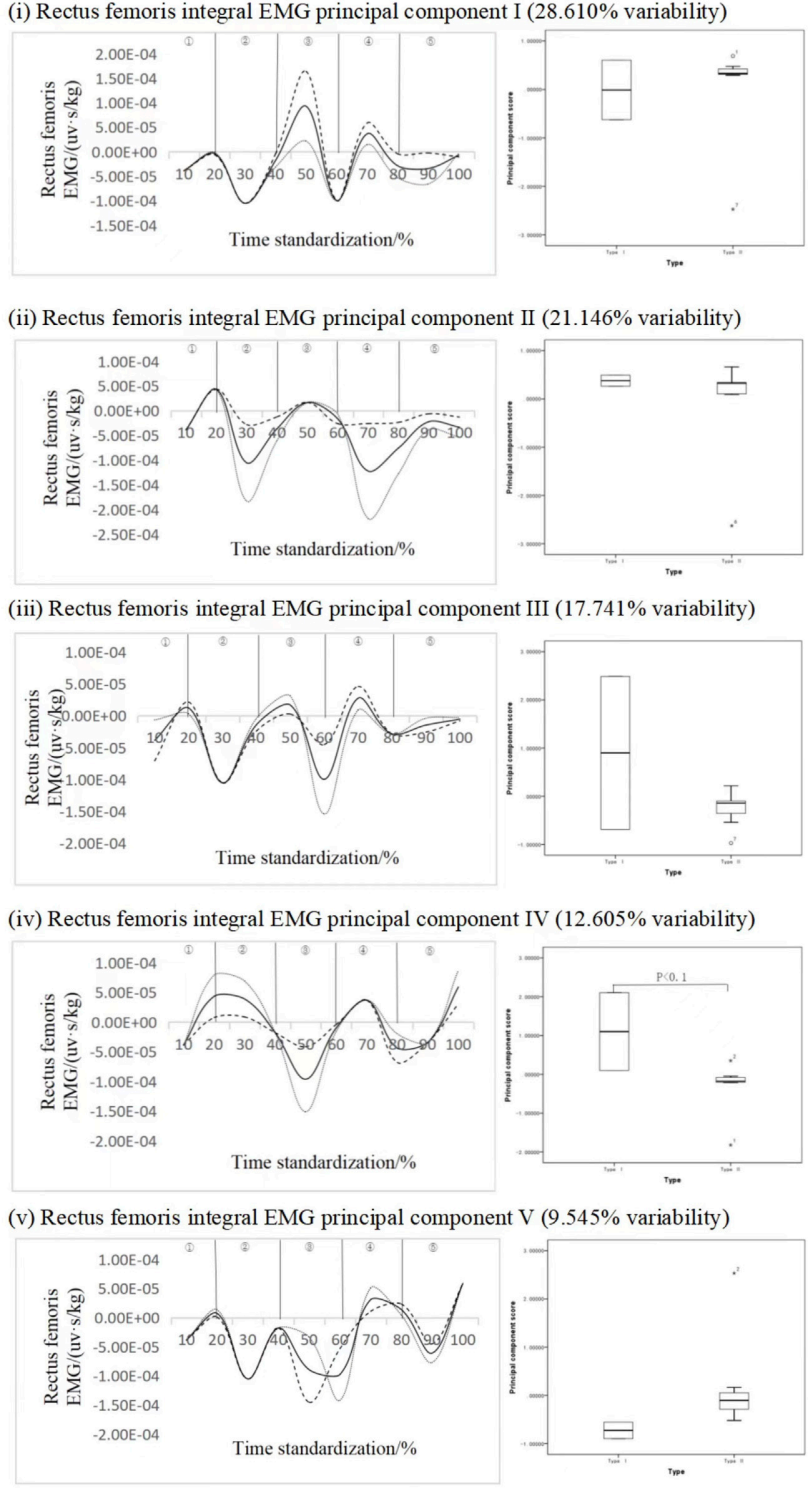
Mean ± weight coefficient of integrated EMG of rectus femoris muscle and principal component scores in different motor development stages.

Figure 8 shows the principal component analysis results of the integrated EMG time series curve of the standing jump femoris rectus muscle. The integrated EMG time series curve can be reduced into five principal components with eigenvalues of 154.492, 114.189, 95.799, 68.067, and 51.543. The variability was 28.610%, 21.146%, 17.741%, 12.605%, and 9.545%, respectively, and the cumulative contribution rate was 89.646%. From the perspective of principal component 1, principal component 2, principal component 3 and principal component 5, there are two fluctuation intervals, and principal component 4 has three fluctuation intervals with significant differences. The results showed that the scores of subjects with standing long jump Ⅰ of principal component IV were higher than those with standing long jump type Ⅱ, and the difference was statistically significant (*p* < 0.1 Figure 4-13iv), ES = 0.51. Therefore, it can be inferred that principal component four can effectively divide different types of movements, and the key force phase occurs in the ascent stage.

#### 4.2.5 Recognition of key phases of triceps brachii

**FIGURE 9:**
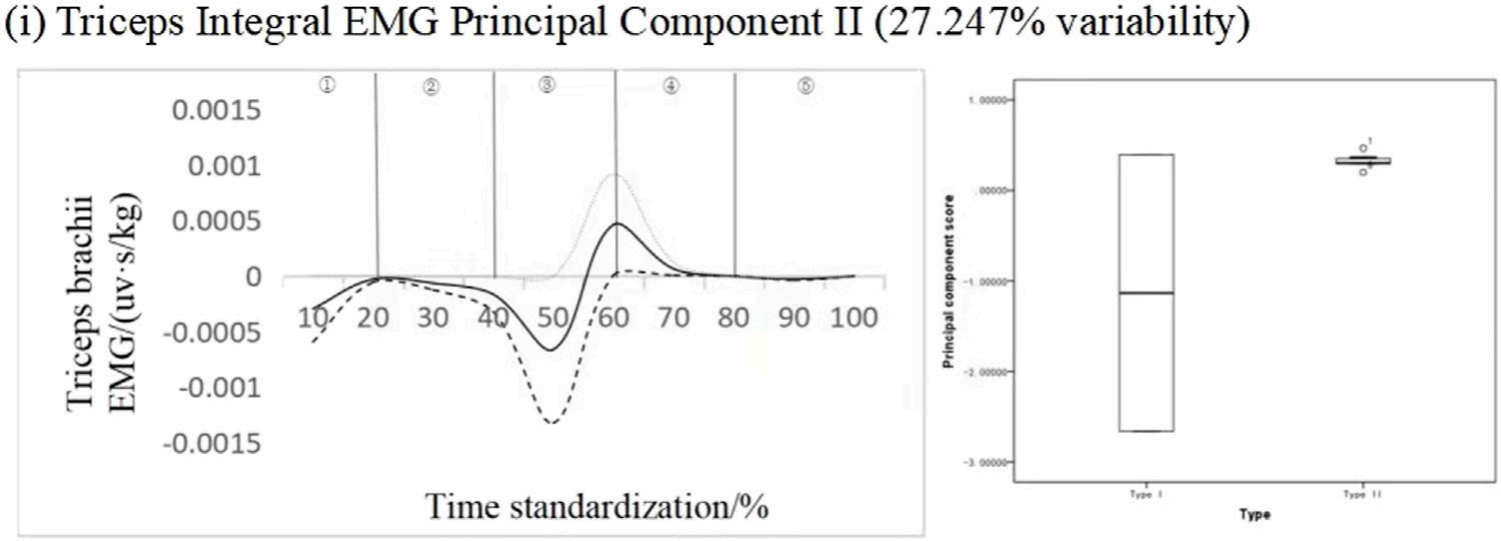
Mean ± weight coefficient of triceps integrated myoelectric and principal component scores in different motor development stages.

Figure 9 shows the results of principal component analysis of triceps integrated EMG time series curve. The integrated EMG time series curve was dimensionically reduced to a principal component with an eigenvalue of 392.445, expressing 72.675% variability and a cumulative contribution rate of 72.675%. The results showed that the scores of all principal components of different types of subjects were not statistically significant. According to the change curve of principal component-1 analysis, there is mainly a variation time interval (30%–70% band), and principal component-1 ΔIEMG% = 99.65%. At the same time, from the principal component score of principal component-1, the principal component score of type I adolescents is lower than that of type II adolescents. It can be seen that the key force phase of this muscle occurs in the air ascent phase.

#### 4.2.6 Trapezius muscle key force phase recognition

**FIGURE 10:**
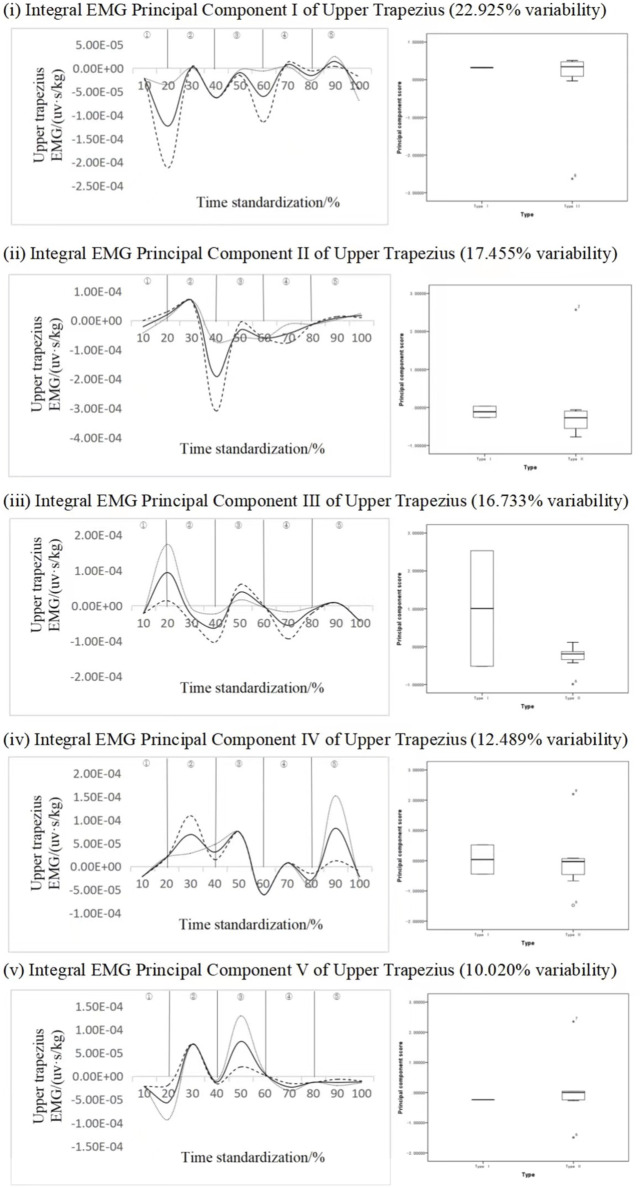
Mean ± weight coefficient of integrated EMG in the upper trapezius muscle and principal component scores in different motor development stages.

Figure 10 shows the principal component analysis results of the integrated EMG time series curve of the upper trapezius muscle. The integrated EMG time series curve is reduced in dimension to five principal components, with the eigenvalues of 123.796, 94.259, 90.361, 67.441, 54.108. They expressed the variability of 22.925%, 17.455%, 16.733%, 12.489% and 10.020%, respectively, and the cumulative contribution rate was 79.623%. The results showed that the scores of all principal components of different types of subjects did not have statistical significance, but the change curves of each principal component analysis showed that there were mainly two to three variation intervals. In particular, the variation characteristics of principal component three are more obvious, but there is no significant, principal component one ΔIEMG% = 94.57%, principal component two ΔIEMG% = 87.51%, principal component three ΔIEMG% = 92.07%, principal component four ΔIEMG% = 91.95%, principal component five ΔIEMG% = 84.59%. It is concluded that principal component one can identify different types of action more effectively, and the key phase occurs in the transition stage from pre-swing to push.

Many previous studies have often elucidated the role of arm movement in the standing long jump by comparing jump performance with and without arm swinging (Feltner M E et al., 1999). However, they did not specifically identify the primary muscle groups affecting the distance in the standing long jump. In this study, through linear regression analysis, we found that the triceps brachii muscle was the most influential muscle affecting the standing long jump distance, followed by the anterior deltoid muscle. Therefore, training to strengthen the anterior deltoid muscle is necessary to effectively improve jump distance. Additionally, this study employed time-series principal component analysis and observed significant differences in the fourth principal component of the rectus femoris muscle. Action recognition scoring and research related to time-series principal component analysis have gradually matured (Xiaolin Liu er ai., 2023) and have been widely applied in studies such as movement development, movement technique analysis (Huijie et al., 2012), sports injury diagnosis (Donoghue et al., 2008; Epifanio et al., 2008), and rehabilitation outcome assessment (Sánchez-Sánchez et al., 2018).

This study identified the fourth principal component of the rectus femoris muscle as a sensitive indicator for action recognition. The variation in the fourth principal component of the rectus femoris corresponds to the transition phases from the late pre-swing to take-off, the airborne ascent phase, and the transition phase from airborne descent to landing. Subjects with Type I standing long jump exhibited higher scores in the fourth principal component of the rectus femoris compared to Type II subjects, indicating greater electromyographic activity in the rectus femoris during Type I jump actions. Through this action process, it was observed that in the late pre-swing to take-off phase, the rectus femoris underwent passive stretching and eccentric contraction, followed by rapid concentric contraction during the take-off extension phase. Comparatively, Type I subjects exhibited deeper knee flexion and a more substantial push-off during the take-off, while Type II subjects typically displayed excessive forward trunk lean, inadequate knee flexion, and lower descent of the center of gravity.

Kockum and Annette, (2015) conducted research on leg muscle force during deep squat jumps and found that the quadriceps femoris muscle is the primary muscle group responsible for deep squat jump actions, which aligns with the results of our regression analysis. By forcefully extending the knees and storing elastic potential energy, a slanting support force is created on the ground, and forward motion is induced by the swinging of both arms. In contrast, Type II subjects exhibited lower knee flexion and less apparent push-off during the initial stages, resulting in reduced jump distance as elastic potential energy is not effectively harnessed.

The next phase is the airborne ascent stage, influenced by the ground’s reaction force. During this stage, both feet leave the ground, initiating leg folding movements, and preparation for leg extension. The role of the rectus femoris muscle in this stage involves knee extension and hip flexion. Type I subjects actively fold their legs during this phase, while Type II subjects may not do so prominently. Insufficient strength in the waist and abdominal area can lead to difficulty in leg folding, resulting in smaller integral electromyographic values in the rectus femoris. The waist area acts as a link between the upper and lower body, and strength in this area is crucial for various aspects of upper and lower body force production.

Lastly, during the final phase from the end of airborne ascent to landing, Type II subjects typically fail to contract their abdominal muscles, preventing active leg extension. Consequently, they are unable to achieve a proper landing posture. Type I subjects extend their legs correctly during this phase, but it is essential to note that excessive leg extension can cause an imbalance in the center of gravity, leading to backward leaning, as consistent with Farhat F’s research (Farhat F et al., 2016). During the landing phase of the standing long jump, bending the knees to absorb the pressure is crucial to protect the knees.

The action selected for this study is the standing long jump, which is a common movement in the youth movement development sequence. This age group is in the golden period of overall development of sports skill patterns, facilitated by the increasing sports-related experience of the subjects in this study, while Type II subjects may lack sports-related experience (Logan et al., 2016). The use of a process-oriented assessment system for the adolescent population has become more widespread in the 2020s, such as the GSGA, which has a wide range of assessments, including static balance, vertical jump, sprinting, catching, jumping, side running, kicking, and hand striking (Barnett et al., 2009a). Although there is no specific sub-scale for skill types, it has received significant attention in the Australian context. However, the assessments obtained through this system do not lead to subsequent training recommendations. Therefore, further research to enhance performance in specific sports is necessary (Barnett et al., 2009b). In summary, the results of this study overall demonstrate the primary muscle groups influencing standing long jump distance and the critical timing of force application for different levels of muscle strength. This could serve as a novel quantitative technique for evaluating muscle scores in action standards in the future.

## 5 Conclusion

1) Judging from the regression analysis of the measured muscles on the distance of the standing jump, the strength of the deltoid muscle is conducive to the improvement of the distance of the standing long jump, which indicates the importance of the strength of the deltoid muscle of the upper limbs.
2) Through time series analysis, it is found that the semitendinous muscle changes from the ascending stage to the descending stage. The rectus femoris, latissimus dorsi and triceps brachii in the ascending stage; The deltoid anterior bundle is in the transition stage from ascending to descending; The force performance of trapezius muscle in the transition stage from pre-swing to push and stretch can be used as the main recognition parameters of standing long jump, which can effectively distinguish different types of movements.

## Data Availability

The original contributions presented in the study are included in the article/supplementary material, further inquiries can be directed to the corresponding author.
